# Looking back, looking forward: lessons from COVID-19 communication measurement, evaluation and learning (MEL)

**DOI:** 10.5365/wpsar.2024.15.1.1056

**Published:** 2024-01-05

**Authors:** Oxana Onilov, Dexin Gong, Kimberly Chriscaden, Jargalan Tsogt, Maria Socorro Melic, Rosemarie Urquico, Anna Biernat, Anna Postovoitova, Lieke Visser, Nancy Wong, Rosemarie North, Olivia Lawe-Davies

**Affiliations:** a Communication Unit, Regional Director’s Office, World Health Organization Regional Office for the Western Pacific, Manila, Philippines.; b Health Emergencies Programme, World Health Organization Regional Office for the Western Pacific, Manila, Philippines.

## Abstract

**Problem:**

Communication is an integral component of an emergency response, including to the coronavirus disease (COVID-19) pandemic. Designing effective communication requires systematic measurement, evaluation and learning.

**Context:**

In the Western Pacific Region, the World Health Organization (WHO) responded to the COVID-19 pandemic by using the Communication for Health (C4H) approach. This included the development and application of a robust measurement, evaluation and learning (MEL) framework to assess the effectiveness of COVID-19 communication, and to share and apply lessons in real time to continuously strengthen the pandemic response.

**Action:**

MEL was applied during the planning, implementation and summative evaluation phases of COVID-19 communication, with evidence-based insights and recommendations continuously integrated in succeeding phases of the COVID-19 response.

**Lessons learned:**

This article captures good practices that helped WHO to implement MEL during the COVID-19 pandemic. It focuses on lessons from the evaluation process, including the importance of planning, data integration, collaboration, partnerships, piggybacking, using existing data and leveraging digital media.

**Discussion:**

Despite some limitations, the systematic application of MEL to COVID-19 communication shows its value in the planning and implementation of effective, evidence-based communication to address public health challenges. It enables the evaluation of outcomes and reflection on lessons identified to strengthen the response to the current pandemic and future emergencies.

## PROBLEM

Communication is an integral component of an infectious disease outbreak response, such as the response to the coronavirus disease (COVID-19) pandemic. (*1*, *2*) Successful communication requires cutting through the informational overload, uncertainty and misinformation to reach a diverse public with information that is accessible, understandable, relevant, credible, trusted, timely and actionable. (*3*, *4*) Communication is essential to support adherence to the public health and social measures (PHSMs) necessary for pandemic management. (*2*, *4*)

Responses to past public health crises have provided robust evidence to support the design and implementation of effective communication interventions; nevertheless, the COVID-19 pandemic presented many new challenges. It was therefore valuable to establish a systematic measurement and evaluation process to ensure that the World Health Organization’s (WHO’s) approach to communication during the COVID-19 response was grounded in the best information and evidence, and was able to evaluate the relevance, effectiveness and efficiency of the communication response. It was also valuable to understand the extent to which communication shaped risk perceptions and contributed to promoting risk-reduction behaviours so that future communication can improve on the successes and address any limitations.

This article describes the measurement, evaluation and learning (MEL) plan used and the lessons identified from the evaluation of WHO’s COVID-19 communication in the Western Pacific Region from 2020 to early 2023. The presentation of MEL findings is beyond the scope of this article.

## CONTEXT

**Fig. 1:**
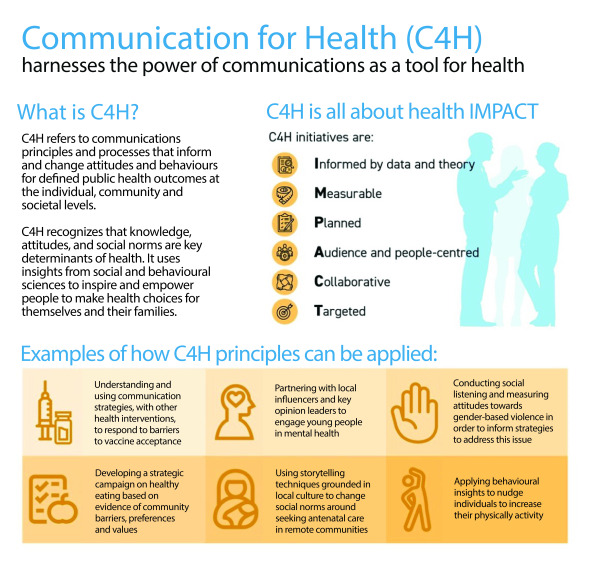
The Communication for Health approach

Since 2019, the WHO Regional Office for the Western Pacific has been using the Communication for Health (C4H) approach (**Fig. 1**), a key component of which is robust and systematic MEL. C4H is a priority for the implementation of *For the Future* – the shared vision for WHO’s work with Member States and partners to make the Western Pacific the safest and healthiest region. (*5*, *6*) The vision recognizes the potential of strategic communication as a public health intervention and a tool for contributing to better health outcomes. The C4H approach brings together a set of principles and practices to help ensure that communication interventions are designed to inform and change attitudes and behaviours in ways that support the achievement of defined public health outcomes. (*7*) MEL is the organizational approach to evaluating C4H. (*8*, *9*) It enables the identification of lessons that are used to fine-tune and adapt strategies, understand what is or is not working, and improve or scale up the effectiveness of communication to help achieve target public health outcomes.

[**insert** Figure 1]

A MEL framework was used during the COVID-19 response to assess the effectiveness of WHO communication in meeting the objectives of informing and changing COVID-19-related knowledge, attitudes and behaviours (KABs) of people across the Region and contributing to the broader goal of reducing transmission and protecting populations from the health impacts of COVID-19.

## ACTION

### Measurement, evaluation and learning

**Fig. 2:**
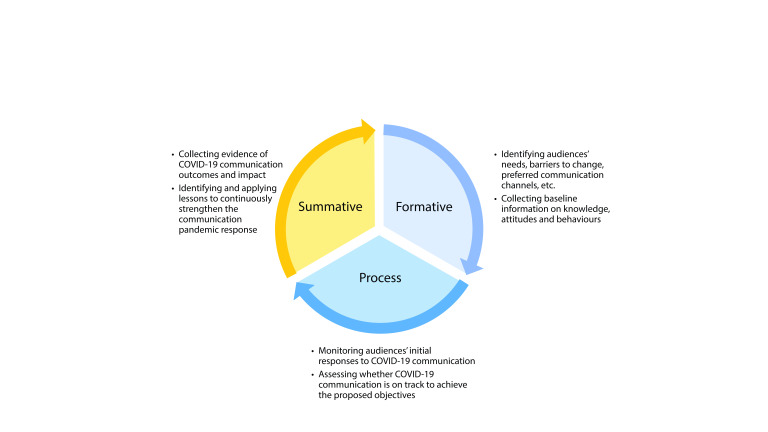
Measurement, evaluation and learning stages

MEL served as a tool to plan and monitor COVID-19 communication interventions progressively from inputs and activities to outputs, outcomes and impact. The MEL plan included metrics and indicators to measure success at formative (before), process (during) and summative (after) evaluation stages, (*9*, *10*) as well as the methods used to generate those indicators (**Fig. 2**).

[**insert** Figure 2]

### Formative MEL

MEL was implemented from the outset, starting with the planning stage of COVID-19 communication. Data from a variety of sources – offline and online, quantitative and qualitative, primary and secondary – were used. Findings collected through these mechanisms were used to plan evidence-informed strategic communication activities as part of the COVID-19 response by Member States, WHO country and regional offices. These data also served as a baseline for KABs to benchmark outcomes related to COVID-19 communication.

In collaboration with partners and global research companies, the communication team at the WHO Regional Office for the Western Pacific conducted two large-scale surveys. The first survey collected evidence on COVID-19 perceptions and behaviours; it was implemented in seven countries in six rounds throughout 2021, 2022 and 2023. The second survey on vaccine confidence was conducted in two rounds across 13 countries in 2021–2022. Focus group discussions in seven countries provided more in-depth responses for some aspects of the quantitative findings (e.g. vaccine hesitancy by age, sex and among people with underlying health conditions). Secondary research data shared by partner agencies were also used to triangulate findings; such agencies included, among others, the United Nations Office for the Coordination of Humanitarian Affairs and the International Federation of Red Cross and Red Crescent Societies, which together with WHO chaired the Asia Pacific Risk Communication and Community Engagement Working Group.

In addition, multisource social listening was routinely employed to track and monitor public opinions expressed online about COVID-19, including emerging concerns, questions and informational needs. This listening involved collecting and analysing native social media and web site analytics, and using existing partnerships and collaborations with media monitoring and social networking platforms to monitor various channels, such as social media, online news, print, broadcasts and podcasts. Although most of the tools used supported analysis of content in multiple languages, media intelligence was interpreted with caution because it skewed towards content produced in English. To respond to this and other data limitations, the team relied on multiple sources to cross-check evidence.

A group of communication professionals at the Regional Office used these data to plan, develop, test and implement COVID-19 communication inputs and activities, hence operationalizing the C4H approach of planned and evidence-based communication.

### Process MEL

Process evaluation was conducted during communication implementation, after the distribution of targeted communication activities, such as social media posts, web site articles, press conferences, media interviews, and online and offline campaigns. This involved monitoring outputs and short-term outcomes to capture message relevance and determine whether progress was being made towards the achievement of objectives.

Lockdown and quarantine measures resulted in dynamic changes to the informational landscape as more people were using digital media for information. WHO’s communication mirrored this shift, with products being disseminated also through social media and the web site. The evaluation at this stage involved analysis of social media and web site analytics, to assess the comments and reactions to social media posts and track the number of visits to WHO’s regional COVID-19 webpage. Analysis of social media comments, for instance, allowed the team to capture and rapidly address misperceptions or misinformation and assess ongoing interest in certain topics.

### Summative MEL

Finally, the team gathered evidence of long-term outcomes and impact after implementation. This included collecting data on knowledge of COVID-19 transmission and protective measures, support for and adherence to PHSMs, and vaccine acceptance among those exposed to WHO advice and those not exposed. Data were also collected on trust in WHO and the role it played in the pandemic.

To capture outcomes and impact, the team relied on the repeat survey data collected through the two large-scale surveys on COVID-19 perceptions and behaviours and on vaccine confidence. The results of each survey round were used to assess the outcomes of WHO communication activities for the preceding months, with results of past rounds used as the baseline. Also, findings were triangulated with secondary research data, where available.

## LESSONS LEARNED

As with the implementation of any activity in the context of COVID-19, evaluating COVID-19 communication has been complex and challenging. Implementing MEL during the pandemic included challenges such as competing priorities for staff time and resources, the vast amount of communication materials distributed through different channels, and the limitations that lockdowns and other PHSMs imposed on traditional data collection (i.e. face-to-face and fieldwork). Reflecting on 3 years of evaluating COVID-19 communication in WHO, this section captures good practices and lessons identified that helped the team to navigate these challenges, assess communication effectiveness and improve through MEL. This list is not exhaustive but is intended to facilitate reflection on how MEL can be adapted to the communication response during crisis situations.

### Integrate data sets

**Fig. 3:**
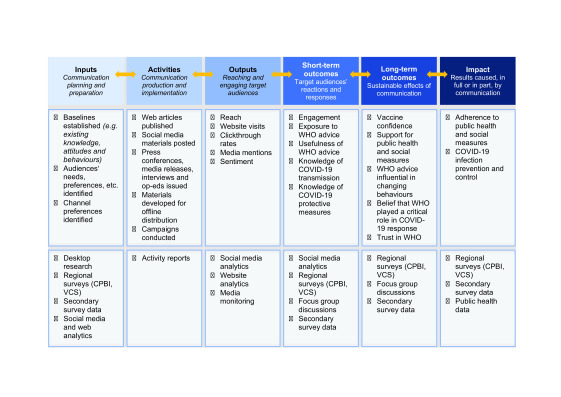
COVID-19 communication MEL indicators and methods

The WHO communication team in the Region used data integration to consolidate disparate but overlapping data sets collected from different sources (**Fig. 3**) into a single data set. This enabled the evaluation of communication as a whole and the measurement of the combined success of different teams. It also ensured a methodical MEL process that provided clear and comprehensive data about what people in the Western Pacific thought, said and did in relation to WHO’s diverse COVID-19 communication.

[**insert** Figure 3]

### Piggyback on other processes

The main purpose of the intelligence gathered through the regional surveys and focus group discussions on COVID-19 perceptions and behaviours and on vaccine confidence was to inform evidence-based and targeted communication for the pandemic response (formative MEL). However, the team was able to include several MEL-related outcomes and impact questions in these research tools to gain insights on the use of, usefulness of and trust in WHO as a source of COVID-19 information, and the role WHO played in the COVID-19 response. The other data collected through these surveys provided the communication team with an understanding of the differences in KABs among those who had seen, read or heard about WHO advice and those who had not.

It was cost effective to add summative MEL questions to these data collection tools that were not necessarily planned to collect evidence of WHO’s communication outcomes and impact. Of particular use was the compilation of a list of possible data mechanisms to which outcome and impact MEL questions could be added during the planning phase.

### Plan MEL early

The success of including MEL questions in the surveys reinforces the importance of planning MEL data sources as early as possible and before implementing communication. Such planning involved clearly defining indicators and selecting the appropriate methods to generate them, as well as mechanisms and roles for data collection and analysis, its uses, reporting schedules and formats. This allowed the team to measure as close to real time as possible, collect realistic and high-quality data, and ensure consistency if changes in reporting responsibilities occurred.

### Use existing data

In addition to primary research, the team used data from the extraordinary amount of research conducted worldwide on COVID-19. Some of the publicly available data collected by other entities met the team’s evaluation needs. Much of the external public health data included information on WHO as a source of COVID-19 information, acknowledging the Organization’s role in disseminating up-to-date information and recommendations.

Secondary data were an effective resource; they were particularly useful owing to the limitations on traditional methods of data collection from PHSMs and because busy communication practitioners did not always have the time and resources to collect data themselves.

### Collaborate across teams

MEL was not the sole responsibility of one evaluator; rather, it was undertaken collaboratively with those planning and implementing different communication activities. There was collaboration between practitioners working on multisource social listening; risk communication and community engagement; content creation and dissemination; outcomes and impact data collection; and the integration, analysis and synthesis of diverse data sources for strategic and actionable insights. This collaboration broke down silos by providing insights into the work of different communication practitioners, contributed to a stronger MEL design and implementation, enhanced data collection and analysis, and produced results that communication practitioners understood and were therefore more likely to use.

### Measure selectively

Prioritizing evaluation increased confidence that resources were being used efficiently and sustainably. When developing the MEL plan, consideration was given to the evaluation mechanisms that could be managed alongside other responsibilities, and to the data collection and analysis that would be available. It is critical to be strategic about what to evaluate, given the limitations in time and resources – it is not necessary or possible to evaluate every single activity. The broader the scope of the evaluation, the more resource-intensive and time-consuming it can be.

### Leverage digital media

The “infodemic” (i.e. too much information, including false or misleading information in digital and physical environments during a disease outbreak) (*11*) presented particular challenges and necessitated activities to combat disinformation, misinformation and rumours in real time. (*12*) The team leveraged the huge increase in visitors to the WHO web site and social media pages, and used available analytics to regularly identify concerns that needed addressing, implement activities designed to debunk specific types of misinformation and disinformation with accurate information, and evaluate audience responses to WHO messages.

### Team up for better design and more MEL

An innovative step in using MEL to understand particular concerns and improve the reach and effectiveness of communication involved teaming up with the social media company Meta (Menlo Park, CA, USA). Through the collaboration, the advice of a specialized digital agency and credits for targeting populations across the Region with advertising campaigns were provided to WHO free of charge. These resources contributed to stronger messaging and more shareable formats that could reach broader and more targeted audiences. For instance, through the analytics of the ad campaigns, the team could identify patterns (i.e. in imagery, format, colours and typography) and the age and sex groups with the highest reach. In turn, this made it possible to tailor future campaigns to the needs and preferences of audiences with whom the previous ad did not resonate.

The collaboration also provided another tool for evaluation: Brand Lift studies. (*13*) These studies measure the effect of a campaign on the target audience’s recall, awareness, motivation and intention, in line with campaign objectives, by comparing two groups – people who have seen the campaign and people who have not. Results of Brand Lift studies were also used to identify areas for improvement and replication.

### Test messages

To improve communication effectiveness, the team determined messaging priorities through social listening and findings from the regional surveys during the MEL planning stage. These were further filtered and drafted into messages that were tested through different methods such as surveys, focus group discussions and viewing panel sessions.

A collaboration with Stickybeak (Auckland, New Zealand), a research and message-testing platform provider, resulted in an innovative approach that used public quantitative chat-based surveys with online target audiences who evaluated various iterations of text, messaging angles and visuals. The findings were used to tweak and refine messages and visuals, and create content that resonated with target audiences, to address their informational needs in the continuously evolving context of the pandemic.

### Build internal MEL capacity

Since the adoption of C4H, WHO in the Western Pacific has trained communication professionals from its regional and country offices in MEL concepts, methods and frameworks. The resulting capacity, in which MEL is recognized as an integral part of the communication cycle, enabled much of the evaluation to be undertaken in-house and helped to ensure that results were delivered in the required time frames and within budget and amid competing priorities.

## Discussion

The COVID-19 pandemic has highlighted the importance of strategic communication as a public health intervention, and the value of applying C4H principles and practices in communication responses to emergencies. Using MEL as part of the C4H approach during the COVID-19 response in the Region has been vital. The Regional Office applied robust MEL at various levels of the planning, implementation and post-implementation phases of COVID-19 communication, and continuously integrated evidence-based insights and recommendations into succeeding phases of the COVID-19 response.

MEL has allowed for the assessment of the effectiveness of WHO communication and a real-time sharing of lessons necessary to adjust plans and strategies for the ongoing crisis response. It encouraged continuous learning and improvement, and thus “supported informed decision-making, encouraged appropriate behaviour change and maintenance among populations, and helped to mitigate adverse health outcomes.” (*14*)

The pandemic provided an intensely dynamic environment in which to implement MEL. Evaluation approaches had to be adjusted to account for the pandemic context. Good practices that helped the communication team of the WHO Regional Office for the Western Pacific to carry out its MEL plan included thinking about and planning evaluations from the outset, using secondary research, applying data integration for comprehensive analysis, piggybacking summative MEL questions onto other data collection tools, leveraging the use of digital media as a real-time source of communication and teaming up with social media platforms. Having internal MEL capacity also allowed for a stronger framework and smoother implementation of MEL.

The team acknowledges that the evaluations undertaken had several limitations. The results are not representative of communities with limited or no access to the internet and mobile networks, and are unlikely to represent the views of vulnerable populations. To respond to this limitation, at least partially, rounds of the regional surveys undertaken in 2023 included face-to-face in-depth interviews with hard-to-reach populations.

It is especially challenging to draw a causal relationship between communication interventions and impact; hence, the team does not make claims of direct results. Impact is multicausal and “communication is just one factor leading to impact.” (*3*) MEL should have evidence that C4H at least contributed to impact. (*15*) The COVID-19 communication MEL evidence showed clear outcomes (e.g. exposure to and trust in WHO advice, knowledge and attitude change, and support for and adherence to protective measures), indicating a direct line from contribution to impact.

Continuing to learn and apply MEL lessons from the pandemic is critical in fully realizing the potential of communication as a public health intervention, including in emergencies, and bringing the Western Pacific closer to achieving the vision, as set out in For the Future, (*6*) of being the safest and healthiest region.
